# The Impact of Distinct Superplasticizers on the Degradation of Concrete Affected by Alkali-Silica Reaction (ASR)

**DOI:** 10.3390/ma16093374

**Published:** 2023-04-25

**Authors:** Andisheh Zahedi, Cassandra Trottier, Yufeng Zhu, Leandro F. M. Sanchez

**Affiliations:** Department of Civil Engineering, Faculty of Engineering, University of Ottawa, Ottawa, ON K1N 6N5, Canada; azahe049@uottawa.ca (A.Z.); ctrot059@uottawa.ca (C.T.); yzhu058@uottawa.ca (Y.Z.)

**Keywords:** alkali-silica reaction (ASR), superplasticizer, alkali content, multilevel assessment, damage rating index (DRI), stiffness damage test (SDT), ASR gel

## Abstract

The effect of two superplasticizers (SPs) with various equivalent (eq.) alkali contents (i.e., with 0.00009% and 4.1% of Na_2_O_eq_, respectively) on the development of an alkali-silica reaction (ASR) was investigated through the use of multilevel assessment. This testing protocol showed promising results for evaluating concrete damage due to ASRs based on mechanical and microscopical testing protocols, specifically the stiffness damage test (SDT) and the damage rating index (DRI). Concrete specimens that incorporated the aforementioned SPs and distinct reactive aggregates (coarse and fine) were manufactured and then stored in conditions that enabled ASR development and were monitored over time. Upon reaching the desired expansion levels of this study, the concrete specimens were prepared for the multilevel assessment. The results show that the SP-incorporated concrete specimens with lower and higher alkali content yielded lower and higher deterioration results, respectively. This clearly confirms that while SP-incorporated concrete that contains SPs with a higher alkali content could increase the risk of ASR deterioration, those SPs with a very low amount of alkali content could act as a mitigation strategy against ASRs. Finally, an investigation into the influence of distinct SPs on the chemical composition of an ASR gel was conducted, which confirmed that the SP with a higher alkali content had the highest potential for further deterioration.

## 1. Introduction

Superplasticizers (SPs) are crucial in today’s concrete industry for the production of modern concrete [1]. Their purposes are to reduce the water-to-cement ratio and enhance the flowability and durability of concrete [2,3]. However, generally, SPs contain sodium and/or potassium, which can increase the alkalinity of the pore solution within the concrete, resulting in alkali-silica reaction (ASR) deterioration. The latter is among the principal sources of early deterioration in concrete, impacting the durability, serviceability, and performance of concrete [4,5]. Very few works have evaluated the effects of various SPs on ASR-induced development, and consequently, the effects of distinct types of SPs on ASR-induced development (i.e., kinetics and induced expansion, damage generation, and propagation, as well as mechanical degradation) remain unknown. In order to appraise ASR-induced deterioration, several tools have been proposed recently; among them, the multilevel assessment protocol, which couples mechanical (i.e., stiffness damage test—SDT) and microscopic (i.e., damage rating index—DRI) tools, proved to be a reliable technique to assess ASR-induced damage in concrete [6,7,8]. Therefore, this work aims to use the multilevel assessment protocol to evaluate the condition of ASR-affected concrete incorporating different types of reactive aggregates (fine vs. coarse), as well as SPs with various alkali content (i.e., none, low and high) and displaying distinct damage degrees (i.e., sound, 0.05%, 0.12%, 0.20%, and 0.30%).

## 2. Background

### 2.1. Alkali-Silica Reaction (ASR) in Concrete

Alkali-silica reaction (ASR) is a deleterious reaction where certain reactive silicates that are found in the aggregates react with the alkali hydroxides (Na^+^, K^+^, and OH^−^) from the concrete’s pore solution, producing a type of alkali-calcium-silica-rich gel (known as the ASR gel) that expands upon water intake, thus cracking the concrete [9,10,11,12,13]. Consequently, such cracking could also accelerate other damage mechanisms in affected elements, such as the corrosion of steel reinforcement bars, freezing and thawing, and/or delayed ettringite formation (DEF). Currently, several of Canada’s and the world’s concrete infrastructures are suffering from ASRs, including dams, bridges, and power plants [13,14,15,16,17,18,19], leading to a significant reduction in the service life of the affected concrete infrastructures. Thus, the maintenance, repair, and replacement costs of the latter considerably increase [20]. Therefore, several mitigation techniques have been proposed to date to prevent ASR-induced damage, including the uses of (a) low alkali cement, (b) nonreactive aggregate, (c) lithium-based admixtures, and (d) supplementary cementitious materials (SCM) [20,21,22,23]. The availability of nonreactive aggregate is relatively low in many locations, while limiting the alkali content of concrete to a severely low level may result in increased energy consumption and, thus, increased cost [24]. On the other hand, despite the very promising results of lithium-based admixtures [25,26], the cost of concrete manufacturing significantly increases, reaching an added cost of approximately 60%, when a sufficient amount of lithium-based admixtures is used to mitigate ASRs [27,28]. Otherwise, the addition of SCMs, such as metakaolin, silica fume, coal fly ash, and blast furnace slag, are amongst the most efficient strategies to mitigate ASRs [21,29,30,31], yet, increases in the water-to-cement ratio could significantly impact the durability properties of the concrete. In order to overcome the above-mentioned drawback of SCMs, superplasticizer (SP) admixtures are generally used in concrete manufacturing [32].

### 2.2. The Use of Superplasticizers in Concrete

SPs are normally used in concrete to improve the workability (i.e., optimizing the flow properties) of concrete mixtures, as well as to facilitate the production of low water-to-cement concrete [33,34]. Therefore, they are considered to be necessary components in advanced concrete technology products, such as self-compacting high-performance concrete in highly congested and low water demand scenarios. SPs include sulfonated naphthalene-formaldehyde condensates (PNS), sulfonated melamine-formaldehyde condensates, modified lignosulphonates and polycarboxylate-ether based molecules (PCE) [33]; this results in the presence of alkali ions (i.e., sodium and/or potassium) in various SPs. Such alkali ions could significantly increase the alkalinity of the concrete’s pore solution, thus increasing the potential for ASR deterioration. According to Uchikawa et al. [35] and Kim et al. [36], the total equivalent (eq.) alkali content (Na_2_O_eq_) of naphthalene-, melamine- or ligno-based SPs is approximately 2–6%, while the polycarboxylate-based SP contains 0.1–1% of Na_2_O_eq_. The organic fraction of the SPs is separated from the pore solution of the concrete within the first few minutes of hydration as the SPs are applied to the mixing water during concrete manufacturing [37], whereas the alkali ions of the SPs remain in the pore solution, increasing its alkalinity [34]. Although the alkali metal contribution of the SPs is minor compared to the amount released by the cement, it can still affect the induced damage caused by ASRs. Very few studies have been performed to understand the effect of SPs on ASR-induced development. Some of these works observed that the addition of an SP could decrease the ASR expansion [38,39,40,41], while others [32,34,42] reported a negative influence on ASR expansion, relatively increasing when comparing the specimens incorporated with SP admixtures to those manufactured without any SPs. These conflicting observations could be attributed to numerous reasons, such as the type and size of the specimen (i.e., mortar bars vs. concrete specimens, associated with various test methods (i.e., ASTM C1260—accelerated mortar-bar method vs. ASTM C 1293—concrete prism test) and the types and alkali content of SPs. As such, Leeman et al. [34] suggested that ASR-induced development is completely dependent on the alkali content of an SP; the higher the alkali content of an SP, the higher the risk of deleterious ASRs. Thus, a higher sodium and potassium content in a particular SP used in a concrete mixture could lead to higher alkali hydroxide concentrations in concrete pore solutions. Such an increase in the alkali content of the pore solution could lead to a faster rupture of the Si–O bonds in the aggregate and accelerate its dissolution and, subsequently, ASR kinetics [34,43].

Accordingly, specimens incorporating a naphthalene-based SP led to an increase in the hydroxide concentration in the pore solution, resulting in an increase in ASR-induced expansion [34], while the impacts of polycarboxylate- and melamine-based SPs are significantly less pronounced and do not result in increased concrete expansion [32,34]. Hence, ASR-induced expansion can be influenced by the type and amount of admixture used in the concrete manufacturing process. Although the above findings are insightful and do suggest that SPs have an important effect on damage due to ASRs, there is currently a lack of studies that quantitatively and systematically assess this type of impact on the microscopic and mechanical properties of concrete incorporating distinct aggregate types and SPs at various ASR-induced expansion levels.

### 2.3. Tools for Assessing Concrete Damaged by ASR

Recently, a number of testing procedures have been developed to assess the condition of damaged concrete and demonstrate the cause and extent of damage (i.e., diagnosis) and potential for further deterioration (i.e., prognosis) of ASR-affected concrete. Among the various tools, a comprehensive multilevel protocol, which consists of mechanical (i.e., stiffness damage test—SDT) and microscopic (i.e., damage rating index—DRI) testing procedures, has been validated as a reliable testing protocol to evaluate the condition of damaged concrete [6,7,12].

#### 2.3.1. Stiffness Damage Test (SDT)

**Figure 1:**
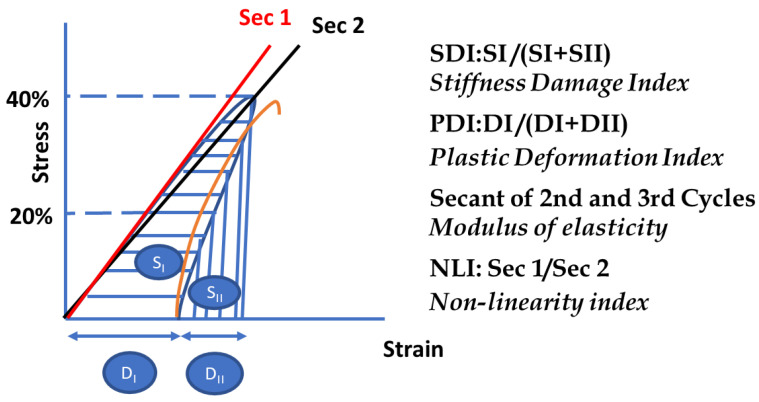
SDT output parameters.

The stiffness damage test (SDT) is a loading/unloading cyclical method in compression that is used as a test protocol to assess the degree of damage in concrete specimens affected by internal swelling reactions (ISRs), such as ASRs [6,7,8]. This method was initially developed by Walsh to correlate crack density and rock specimens’ stress/strain relationship [44,45], after which it was used to appraise ASR-induced damage in affected concrete by Crisp et al. [46,47]. Later, Sanchez et al. [44,45] optimized this method by modifying the test procedure; the authors [44,45], therefore, proposed to use cycles of five loading/unloading regimens at 40% of the compressive strength of sound concrete (i.e., design/28-day strength) under a loading rate of 0.10 MPa/s. Furthermore, Sanchez et al. [44,45] have also adapted indices as the output parameters of the test procedure to reliably assess ASR damage in concrete. The ratio of dissipated energy-to-total energy is, therefore, the stiffness damage index (SDI = SI/(SI + SII)), whereas the ratio of plastic deformation-to-total deformation is the plastic deformation index (PDI = DI/(DI + DII)) over the five cycles, as per [44,45] and illustrated in Figure 1. Moreover, the modulus of elasticity, as calculated by the average secant modulus of the 2nd and 3rd cycles along with the Non-Linearity Index (NLI), which is the secant moduli of half over the maximum load in the first cycle and Sec 1/Sec 2, are some of the other SDT outcomes used to detect the extent and orientation (i.e., NLI is higher/lower than one for cracks that are oriented perpendicular/parallel to the loading direction as per Crisp et al. [46,47]) of ASR-induced damage, respectively. A full review/history of the SDT as a testing protocol for appraising the condition of ASR-affected concrete can be found in [44,45].

#### 2.3.2. Damage Rating Index (DRI)

**Figure 2:**
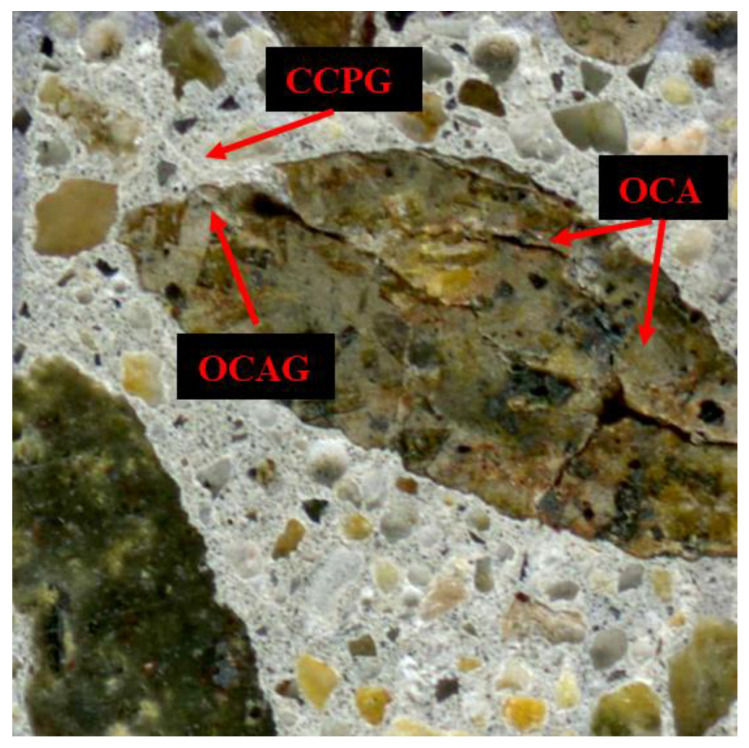
Some of the damage features in the 1 cm^2^ concrete section.

Grattan-Bellew and Danay [48,49] developed a semi-quantitative microscopic procedure, the damage rating index (DRI), to appraise the extent of damage in ASR-affected concrete, where the DRI number generally increases with expansion. As such, distinct petrographic/damage features (Figure 2) are counted in 1 cm by 1 cm squares that are drawn on the surface of polished concrete specimens with the aid of a stereomicroscope at 15–16× magnification [50]. Those petrographic/damage features are then multiplied by nonarbitrary weighting factors, as proposed by Villeneuve et al. [51], where the closed cracks in aggregates (CCAs) have a weighting factor of 0.25, and the opened cracks in aggregates (OCAs), opened crack with reaction product in aggregates (OCAGs), and disaggregate/corroded aggregate particles (DAPs) have a weighting factor of 2, and the coarse aggregate debonded (CAD) and cracks in cement paste (CCP), as well as cracks with reaction product in cement paste (CCPG), have a weighting factor of 3. Thus, balancing the relative importance of a given distress feature with respect to the associated overall distress mechanism, such as ASRs, occurs while reducing the variability among the operators. As such, the given weighting factor of a crack with or without reaction product (i.e., hereafter referred to as ASR gel for practical purposes) is identical since the identification and interpretation of such distress features might vary among petrographers [52]. A low importance was given to the closed crack in the aggregates (CCA, i.e., a weighting factor of 0.25) since such cracks are less likely to be associated with ASRs (e.g., weathering or aggregates manufacturing). Otherwise, a weighting factor of 2 for the open cracks in the aggregate particles with or without gel (OCA and OCAG, respectively) is attributed to the ASR-induced development and, thus, given higher importance. Finally, a weighting factor of 3 is given to the cracks in a cement paste with or without gel (CCP and CCPG, respectively), indeed reflecting a more severe and progressed level of deterioration in ASR-affected concrete. Finally, the obtained weighted counts are normalized to a 100 cm^2^ area, resulting in the DRI number, which is used for comparative purposes, where a higher DRI number corresponds to a higher degree of damage in affected concrete [6]. Moreover, a surface of at least 200 cm^2^ should be appraised per member for real structures in order to provide a statistically significant DRI result and, thus, a more reliable appraisal of the extent of the damage.

## 3. Scope of Work

Superplasticizers (SPs) are mainly used to enhance the performance of concrete in its fresh and hardened states, yet their influence on ASR-induced development remains unknown in terms of damage, as captured through the multilevel assessment. This study, therefore, aims to perform a multilevel assessment of concrete by incorporating distinct reactive coarse and fine aggregates (i.e., New Mexico: polymictic gravel and Texas: polymictic sand, respectively) while varying the type of SPs used via low–high alkali content. The specimens were manufactured in the laboratory and were monitored over time until the desired expansion level (i.e., %ℓ/ℓ) was achieved (i.e., 0.05%, 0.12%, 0.20%, 0.25%, and/or 0.30%). The ASR-induced expansion and kinetics of conventional and SP-incorporated concrete were compared, followed by damage development, in terms of mechanical degradation and crack generation and propagation.

## 4. Material and Methods

### 4.1. Materials and Mixture Proportions

A total of one hundred concrete cylinders, 100 mm in diameter and 200 mm in length, were fabricated in the laboratory with a design compressive strength of 45 MPa. Highly reactive fine and coarse aggregates (i.e., Texas and New Mexico, respectively) were combined with nonreactive fine (i.e., Laval) and coarse aggregates (i.e., Québec, respectively), and these were used for concrete fabrication. The various aggregates used in this work, along with their lithological composition, are listed in Table 1. All concrete mixtures were produced with general-use Portland cement (CSA Type GU) with an alkali content (Na_2_O_eq_) of 0.88%. Additionally, to accelerate ASR development, the total alkali content of both mixtures was increased to 1.25 percent Na_2_O_eq_ by cement mass. Moreover, two different superplasticizers with low to high alkali content (i.e., Admixtures 1 and 2, respectively) were selected for this study; their chemical compositions are presented in Table 2. The dosages of Admixtures 1 and 2 were 65 mL and 580 mL per 100 kg of cementitious material, respectively, as recommended by the manufacturers. The alkali content of the admixtures was not considered in the concrete mixtures proportioning so that we could evaluate the influence of the distinct admixtures. Moreover, only Admixture 1 was used in the concrete made with the reactive coarse aggregate (NM), while both admixtures (i.e., Admixtures 1 and 2) were used for the concrete incorporating TX sand (Table 3). For comparison purposes, the w/c ratio was kept constant for all mixtures in this study (w/c = 0.37). Particle size distributions of the fine and coarse aggregates were kept constant, following the specified sizes, as per ASTM C1293 [53].

### 4.2. Fabrication of Concrete Specimens

According to ASTM C 192 [56], all concrete specimens were demolded after 24 h and moist-cured (i.e., 20 °C and 100% RH) for an additional 24 h. Holes of 8.5 mm in diameter by 19 mm in length were drilled at both ends of all concrete specimens, and stainless-steel gauge studs were glued with a fast-setting cement slurry for the axial expansion measurements, left to moist-cure for another 24 h. The initial length readings were then taken, and all concrete specimens were placed in air-tight 22 L containers under a film of water and lined with a damp cloth, after which the specimens were stored in conditions that enable ASR development in the laboratory (i.e., 38 °C and 100% RH) and their lengths were monitored over time. Upon reaching the given expansion levels (i.e., 0.05%, 0.12%, and 0.20% for both NM and TX concrete specimens and 0.25% and 0.30% for the NM and TX concrete specimens, respectively), the specimens were removed from the ASR-enabling conditions and prepared for testing. Control specimens were also fabricated using the same mixture proportions without the use of the admixtures for comparative purposes. The latter was placed in sealed buckets and stored in a cold chamber (i.e., 12 °C) in order to stop ASR development, as per [57].

### 4.3. Experimental Procedures

#### 4.3.1. Stiffness Damage Test (SDT)

The loading level for conducting the SDT was initially determined through the designed compressive strength test, which was performed on two undamaged specimens (i.e., control). As ASRs may develop in the control specimens using the same mixture proportions [57], the conventional 28-day compressive strength (i.e., ASTM C 39 [58]) test could not be used for the control concrete specimens; therefore, those specimens were wrapped and placed at 12 °C for a 47-day period, which represents the equivalent maturity to samples cured for 28 days at 20 °C, as per ASTM C 1074 [59]. Next, both end surfaces of all concrete cylinders were mechanically ground to eliminate any interference from the gauge studs used for monitoring length change. Three specimens from each concrete mixture at the selected expansion levels were used for the SDT, as per the procedure proposed by Sanchez et al. [44,45], and were subjected to five cycles of loading/unloading at a controlled loading rate of 0.10 MPa/s up to a maximum load of 40% of the compressive strength of the control specimens. It is worth noting that according to CSA23.2-14C [60], and as per Sanchez et al. [44], all concrete specimens were reconditioned for two days in the moist curing cabinet to preserve the water in the concrete (to reduce test variability) prior to the STD.

#### 4.3.2. Damage Rating Index (DRI)

For each expansion level, two concrete cylinders were cut in half lengthwise with a masonry saw equipped with a diamond blade and were polished with the aid of a mechanical hand polisher with grits/abrasives of 30 (coarse), 60, 140, 280 (80–100 microns), 600 (20–40 microns), 1200 (10–20 microns), and 3000 (4–8 microns) prior to the microscopic assessment. Grids of 1 cm by 1 cm were drawn on the surface of the polished concrete specimens, representing the field of view at 15–16× magnification while using a stereomicroscope. The damage features (i.e., cracks) down to 1 mm in size were counted in each grid. The weighted counts (using the proposed weighting factors by Villeneuve et al. [51]) were then normalized to 100 cm^2^, which represents the DRI number.

## 5. Results

### 5.1. The Development of ASR Damage through Expansion

**Figure 3:**
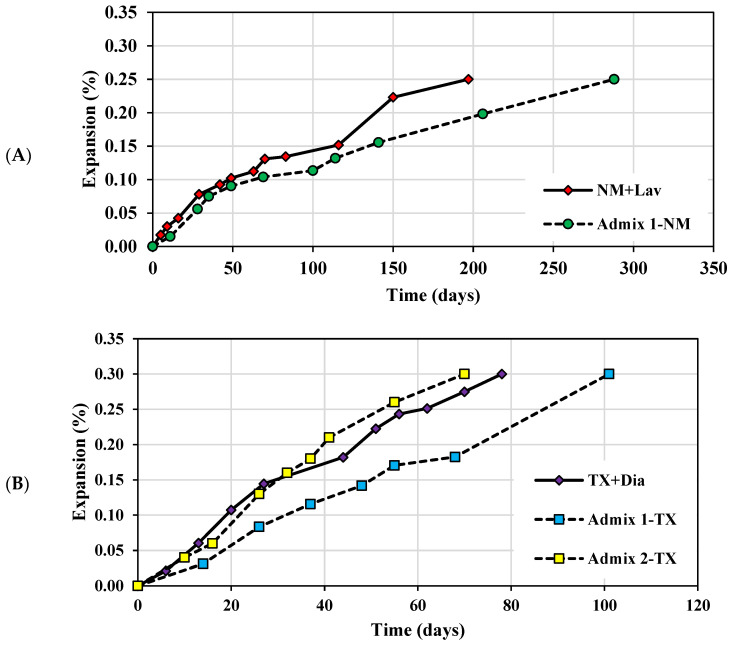
ASR expansion as a function of time for concrete specimens incorporating Admixtures 1 and 2 and made of (**A**) reactive coarse aggregate (NM) and (**B**) reactive sand (TX).

The average expansion levels as a function of time in the ASR-affected concrete specimens made with a reactive coarse aggregate (i.e., New Mexico—NM) and reactive sand (i.e., Texas—TX) are illustrated in Figure 3A,B, respectively. The standard deviation of the NM and TX mixtures ranges from 0.01 to 0.04% and 0.02 to 0.04%, respectively. Overall, the expansion level increased with time when all concrete specimens reached the expansion levels selected for this study (i.e., 0.25% and 0.30% for the NM and TX, respectively).

Generally, the New Mexico (NM + Lav) concrete mixtures follow a parallel trend, where the conventional concrete (CC) and Admixture 1-incorporated concrete mixtures reached 0.11% and 0.10% at 63 and 69 days, respectively. Later, the Admixture 1-incorporated concrete was followed by a decrease in the expansion rate, reaching 0.25% of expansion at 288 days, while 197 days were required for the conventional NM + Lav concrete to achieve the same level of expansion. On the other hand, the Texas (TX + Dia) concrete mixtures present faster kinetics when compared to the reactive coarse aggregate. A parallel trend is observed for the Admixture 2 and CC-incorporated concrete mixtures, both reaching 0.30% of expansion after 70 and 78 days, respectively, while 101 days were required for the Admixture 1-incorporated concrete, thus presenting a lower rate of expansion. This clearly shows that both mixtures incorporating Admixture 1 presented lower expansion levels when compared to CC, while Admixture 2 increased the expansion level of the Texas concrete specimens.

### 5.2. Mechanical Damage via the Stiffness Damage Test (SDT)

**Figure 4:**
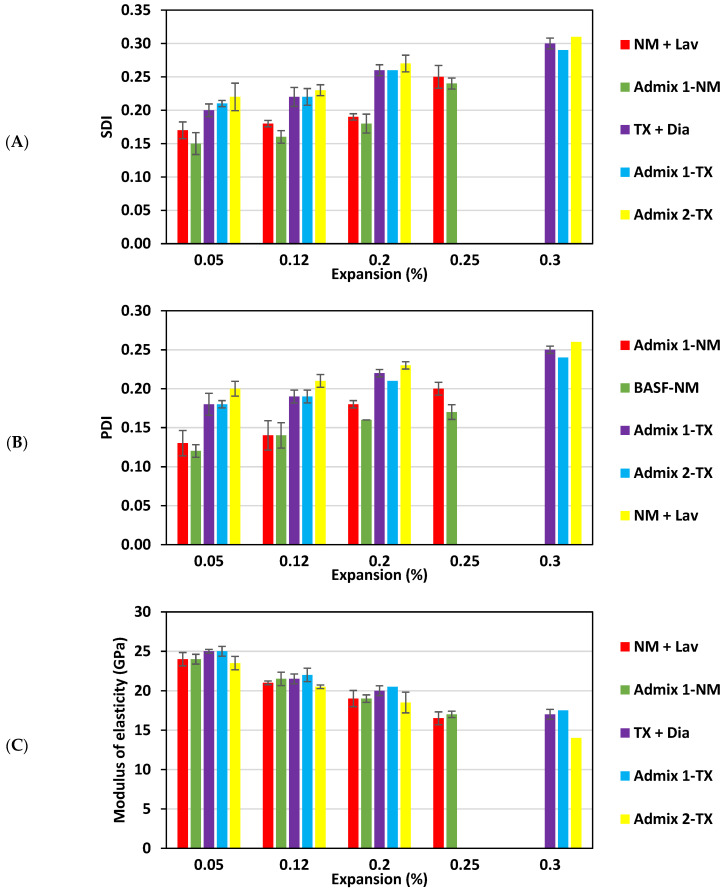

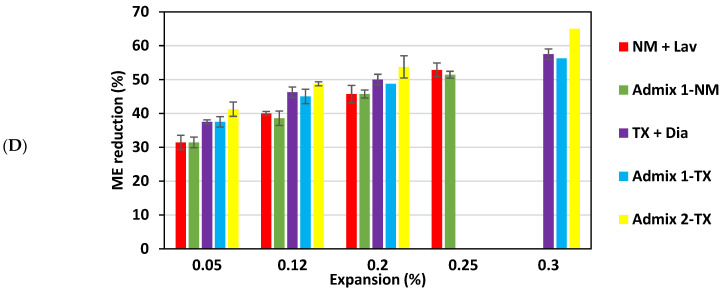
Stiffness damage test results: (**A**) SDI, (**B**) PDI, (**C**) modulus of elasticity (GPa), and (**D**) modulus of elasticity reduction (%).

The maximum load used for the SDT was 18.8 MPa for a compressive strength of 47 MPa. Figure 4 presents the SDI, PDI, and modulus of elasticity (ME) for each expansion level, where both SDI and PDI generally increase with expansion, whereas a decrease is observed for the ME.

The SDI values (Figure 4A—standard deviations presented in the plot) range from 0.17 to 0.25 and from 0.15 to 0.24 for the NM + Lav mixtures without and with Admixture 1, respectively, whereas the range of SDI values for the TX + Dia are 0.20–0.30, 0.21–29, and 0.22–0.31 without and with Admixtures 1 and 2, respectively. Likewise, PDI values of 0.13–0.20 and 0.12–0.17 were obtained for the NM + Lav concrete samples without and with the Admixture 1, respectively, while ranges of 0.18–0.25, 0.18–0.24, and 0.20–0.26 were found for the TX + Dia without and with Admixtures 1 and 2, respectively (Figure 4B—standard deviations added to the plot).

Furthermore, a decrease in modulus of elasticity (ME) from an undamaged/sound CC incorporating NM (i.e., 35 GPa) and TX (i.e., 40 GPa) is presented in Figure 4C (with their standard deviations). The ME values of CC-NM decreased from 24 GPa at 0.05% of expansion to 16.5 GPa at 0.25% of expansion, while Admix 1-NM decreased from 24 GPa to 17 GPa for the same given expansion levels, respectively. This clearly represents an overall ME reduction range of 31–53% (Figure 4D—including standard deviations). Moreover, the ME values of CC-TX, Admix 1-TX, and Admix 2-TX decrease as expansion increases (i.e., from 0.05% to 0.30% of expansion) from 25 GPa to17 GPa, from 25 GPa to 17.5 GPa, and from 23.5 GPa to 14 GPa, respectively, while presenting ME reductions of 38–58%, 38–57%, and 41–65%, respectively.

### 5.3. Microscopic Analysis via the Damage Rating Index (DRI)

**Figure 5:**
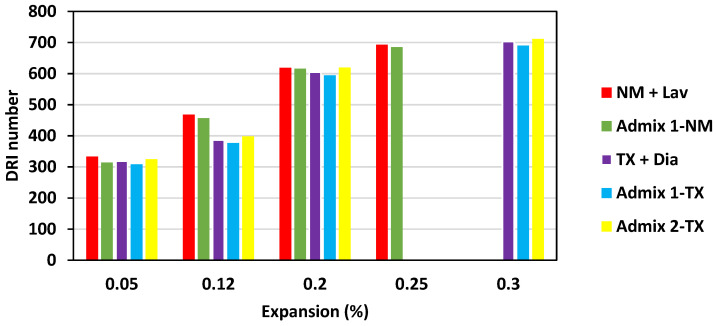
DRI value as a function of expansion for all concrete specimens in this study.

Figure 5 illustrates the DRI numbers for all concrete mixtures incorporating the reactive coarse and fine aggregates (i.e., New Mexico—NM and Texas—TX, respectively). Evidently, the DRI number increases with expansion; hence, the DRI captures the damage level. As such, the DRI number of the NM + Lav and Admix 1-NM specimens (i.e., from 0.05% to 0.25% of expansion) ranges from 330–695 and 310–685, respectively, whereas the TX + Dia, Admix 1-TX, and Admix 2-TX concrete specimens (i.e., from 0.05% to 0.30% of expansion) demonstrate DRI values ranging from 315–705, 305–690, and 325–715, respectively.

## 6. Discussion

### 6.1. The Impact of Distinct Superplasticizers on ASR Damage in Concrete

#### 6.1.1. Expansion and Kinetics

Overall, each concrete mixture presented an increase in expansion with time, yet differences were indeed observed with regard to the induced expansion and kinetics at the same given time. Evidently, the kinetics were different for concrete made with a reactive fine or coarse aggregate (i.e., TX vs. NM), as previously observed by [6,61]. However, both concrete samples incorporating Admixture 1, regardless of the aggregate type (i.e., fine or coarse aggregate), achieved the same level of expansion as their companion CC yet at a later day (Figure 3). Conversely, the concrete specimens that incorporated reactive sand and Admixture 2 show slightly slower expansion kinetics compared to CC at the beginning of the reaction (e.g., up to 0.14% ± 0.01 at 35 days—Figure 3B), after which the specimens made with Admixture 2 surpass the CC samples (e.g., achieving 0.30% expansion at 78 and 70 days, respectively).

Indeed, the difference in the alkali content in the admixtures might have contributed to the expansion levels observed, where Admixture 1 had a significantly low alkali content of 0.00009% Na_2_O_eq_ compared to Admixture 2 with a 4.1% alkali content; the higher the alkali content, the greater the expansion. In this regard, Esfahani et al. [38] and Gillott et al. [39] have previously observed that SPs with low alkali content were able to reduce the ASR-induced expansion in affected concrete. All of the above, therefore, attests that SPs with significantly low alkali content can be an effective measure to mitigate ASR-induced development, as per Leemann et al. [34] and some guidelines (e.g., [62]). On the other hand, as per Flaviana et al. [32], the higher the alkali content in an SP used in the mixture, the higher the risk of damage due to ASRs. This high alkali content in distinct SPs can result in an increase in alkali hydroxide concentrations in the pore solution [34,63]. As previously stated, the increase in hydroxide concentration in concrete can result in a faster breakdown of Si–O bonds in the aggregate and lead to an increase in the ASR kinetics [34,43]. It is worth noting that even though the contribution of alkali content provided by SPs is significantly lower compared to the amount released by cement (e.g., the Na_2_O_eq_ provided by Admixture 2 and the cement paste is around 200 gr and 3800 gr, respectively, as per this study), the instant availability of sodium and potassium released by SP can impact the hydroxide concentration of a pore solution at an early age, resulting in an increase in ASR-induced expansion [34,37].

#### 6.1.2. Measuring Deterioration

Distinct superplasticizers can considerably affect ASR-induced damage development (i.e., damage generation and propagation, as well as mechanical degradation) [32,34,38,39,40,41,42]. Therefore, the extensive testing performed in this study helped to better understand the correlation between different SPs and ASR-induced damage development as a function of the expansion level. Hence, the coupling of the ASR-induced microcracking and mechanical property losses will be discussed in the following subsections.

- Microscopic Assessment

**Figure 6:**
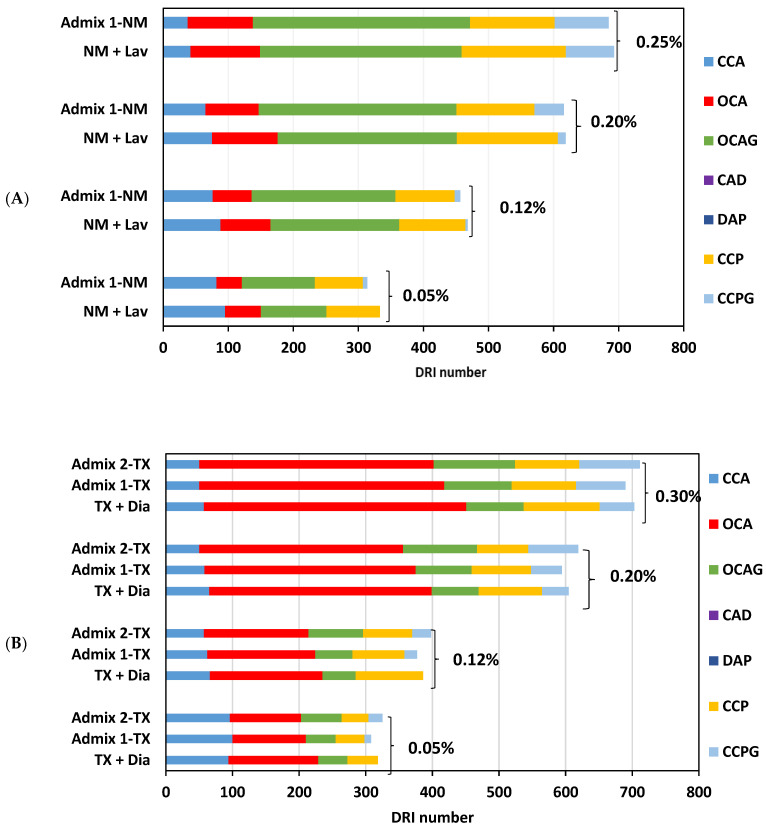
DRI charts for concrete specimens incorporating (**A**) NM coarse aggregate and (**B**) TX sand aggregate.

The microscopic assessment carried out on various concrete samples in this work (Figure 5) demonstrates that, although all the DRI numbers are in the same range, the Admixture 2- and 1-incorporated specimens have slightly higher and lower DRI numbers compared to the CC samples, respectively. As such, the NM + Lav and Admix 1-NM specimens exhibited a DRI number of 700 and 685 at 0.25% expansion. Likewise, at 0.30% expansion, TX + Dia, Admix 1-TX, and Admix 2-TX displayed DRI numbers of 700, 685, and 715, respectively. In order to better visualize the microscopic difference between the distinct specimens in this work, Figure 6 illustrates extensive DRI bar charts, showing all the identified petrographic features of the distinct concrete specimens as a function of ASR development. All mixtures display an increase in the number of open cracks in the aggregates without and with gel (i.e., OCA and OCAG, respectively) and the number of cracks in the cement paste without and with gel (i.e., CCP and CCPG, respectively) with expansion. Although the final DRI values of all the concrete specimens are quite similar at each expansion level, the most notable differences in the distress features between the conventional concrete and distinct SP-incorporated concrete specimens are open cracks in the aggregates without and with gel (OCA and OCAG—red and green bar, respectively) and the cracks in the cement paste without and with gel (i.e., CCP and CCPG—orange and light blue bar, respectively).

**Figure 7:**
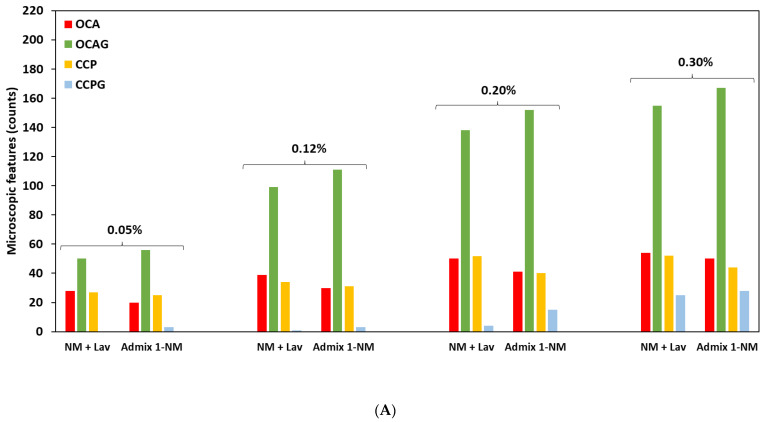

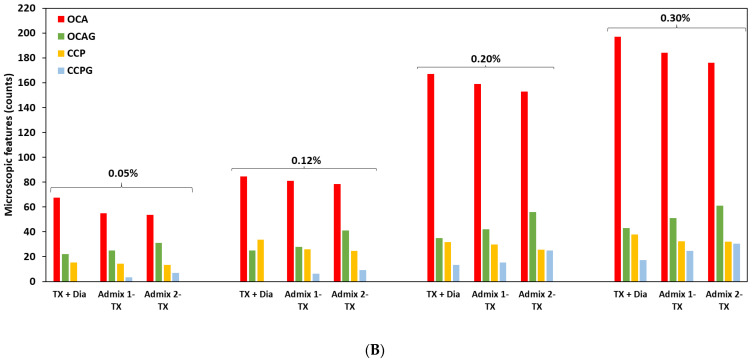
Counts of microscopic features in concrete specimens incorporating (**A**) NM coarse aggregate and (**B**) TX sand aggregate.

In order to better demonstrate the above differences, Figure 6 shows the features that were appraised in an absolute (counts) manner, without the application of any weighting factors through the use of an extended DRI version, as per Sanchez et al. [64] (Figure 7). When analyzing Figure 7, one observes the following distress features ((i.e., cracks) associated with ASR and counted through the DRI) in the SP-incorporated concrete mixtures: (A) a higher number of opened cracks in the aggregates without ASR gel (OCA—red chart in Figure 6 and Figure 7) was found in the conventional concrete mixtures (i.e., without admixtures) compared to SP-incorporated concrete, e.g., the number of open cracks in the aggregate (OCA) found in each concrete specimen at the highest expansion level of this study (i.e., 0.25% and 0.30% for reactive coarse and fine aggregate, respectively) is as follows: 54 for CC-NM vs. 50 for Admix 1-NM, 197 for CC-TX vs. 184 and 175 for Admix 1-TX and Admix 2-TX, respectively; (B) a higher number of cracks in the cement paste without ASR gel (CCP—orange chart in Figure 6 and Figure 7) was observed in the CC samples when compared to the SP-incorporated specimens; for instance, the number of cracks in the cement paste (CCP) found in each concrete mixture at the highest expansion level in this work (i.e., 0.25% and 0.30% for reactive coarse and fine aggregate, respectively) is as follow: 52 for NM + Lav vs. 44 for Admix 1-NM and 38 for TX + Dia vs. 33 and 32 for Admix 1-TX and Admix 2-TX, respectively; (C) conversely, the SP-incorporated mixtures displayed a higher number of open cracks in the aggregates with ASR gel (OCAG—green chart in Figure 6 and Figure 7): 167 for Admix 1-NM vs. 155 for NM+ Lav and 61 and 51 for Admix 2-TX and Admix 1-TX, respectively vs. 43 for TX + Dia, was observed at the highest expansion level in this study (i.e., 0.25% and 0.30% for reactive coarse and fine aggregate, respectively); (D) similarly, the mixtures with SPs presented a higher number of cracks in the cement paste with ASR gel (CCPG-light blue chart in Figure 6 and Figure 7); for example, 28 for Admix 1-NM vs. 24 for NM + Lav and 31 and 25 for Admix 2-TX and Admix 1-TX, respectively vs. 17 for TX + Dia at the highest expansion level in this study (i.e., 0.25% and 0.30% for reactive coarse and fine aggregate, respectively). In summary, in the case of OCA and CCP, the following relation is true: CC > Admixture 1 > Admixture 2, whereas OCAG and CCPG follow the opposite order: Admixture 2 > Admixture 1 > CC. The increase in the number of cracks with ASR gel in the aggregate and cement paste in the mixtures incorporating distinct SPs could be due to the chemical composition of those admixtures (i.e., especially the alkali content), which might significantly change the chemical composition of the ASR product. This will be thoroughly discussed in the next section.

- Mechanical Property Losses

When analyzing the graphs in Figure 4, a decreasing trend for the mechanical properties of all the concrete families as a function of ASR-induced development is clearly observed. This is in accordance with the observations made by various authors (e.g., [6,61,65,66]), in which the greater the development of an ASR, the greater the mechanical property loss. As such, according to the research, concrete affected by ASRs experiences a considerable decrease in tensile strength (as low as 65%) and modulus of elasticity (as low as 50%) at low to moderate levels of expansion (between 0.05% to 0.12%) [6]. On the other hand, compressive strength is only significantly affected at high (0.20%) or very high (0.30%) levels of expansion [6,7]. The stiffness of the aggregate particles is a crucial factor in influencing the reduction in the modulus of elasticity in ASR-affected concrete. ASR cracking typically arises at the submicroscopic level within the aggregate particles; therefore, a decrease in stiffness can be observed even at low expansion levels (i.e., 0.05%). Furthermore, tensile strength experiences a significant reduction in the early stages of ASR (i.e., from 0.05% to 0.12%) due to the dependence of porous materials, such as concrete, on the existence and size of cracks, as per fracture mechanics principles. The peaks of “stress concentration” are created at the tips of ASR-induced cracks in concrete under tension, resulting in crack propagation and eventual failure [6]. Conversely, the compression failure mechanism is more ductile, characterized by the failure of the “cement paste,” with cracks initially forming at the interfacial transition zone (ITZ) at various locations and later propagating to the bulk of the cement paste, ultimately leading to system instability and failure [67]. Therefore, since ASR-induced cracks form primarily within the aggregate particles and extend to the cement paste at later stages, ASR-induced development causes a gradual loss in the compressive strength in the affected concrete [6].

Similar to the microscopic assessment regarding the SDT outputs (i.e., SDI, PDI, and ME) displayed in Figure 4, although all the mechanical property losses of most of the concrete specimens (i.e., regardless of aggregate types) are within the same range), Admixtures 2 and 1 present higher and lower mechanical property losses (i.e., SDI, PDI, and ME) when compared to the CC specimens, respectively. As such, while the NM + Lav specimens present a 5% and 15% higher SDI and PDI, respectively, compared to the Admix 1 -NM specimens, the Admix 2-TX specimens demonstrate a 6% and 4% higher SDI and a 9% and 5% higher PDI compared to Admix 1-TX and TX + Dia, respectively, at the highest expansion level in this study (i.e., 0.25% and 0.30% for the NM- and TX-incorporated specimens, respectively). Likewise, when analyzing Figure 4C,D, one sees that the Admix 1-TX concrete exhibited an 18% and 20% higher ME compared to the TX + Dia and Admix 2-TX specimens, respectively, at the highest expansion level in this study (i.e., 0.30%). Yet, when comparing the ME gathered from NM + Lav and Admix 1-NM, only a 4% higher ME was obtained. The above discussion once again confirms that while SPs with a much lower alkali content (i.e., Admixture 1 in this study with alkali content of 0.00009% Na_2_O_eq_) could slightly reduce the development of ASR damage, those admixtures with a high alkali content (i.e., Admixture 2 in this study, with an alkali content of 4.1% Na_2_O_eq_) could increase the mechanical property losses in ASR-affected concrete.

Moreover, A two-way analysis of variance (ANOVA with a confidence level of 95%) was conducted as a function of ASR-induced expansion against SDT outcome (i.e., SDI, PDI, and ME) to evaluate the statistical significance of these results. According to Table 4, all the SDT outcomes are statistically significant for all the concrete specimens in this work, with all the “F values” being higher than the “Fcritic”, and the “*p* values” being lower than 0.05. This, therefore, attests to the “diagnostic” nature of the SDT to appraise the mechanical property losses in the distinct superplasticizer-incorporated concrete specimens used in this work.

- Summary

The aforementioned results gathered through the mechanical testing and microscopic assessment clearly highlight the impact of distinct SPs on ASR damage, whereby the concrete specimens made with Admixtures 2 and 1 experienced slightly greater and lesser damage, respectively, when compared to conventional concrete (CC). As was thoroughly discussed in the previous section, these differences among the various superplasticizers used in this study could be directly attributable to the alkali chemical composition of those admixtures, as per Leeman et al. [34] and Flaviana et al. [32]; Admixtures 1 and 2 had an alkali content of 0.00009% and 4.1 ± 0.1% of Na_2_O_eq_, respectively (Table 2).

**Figure 8:**
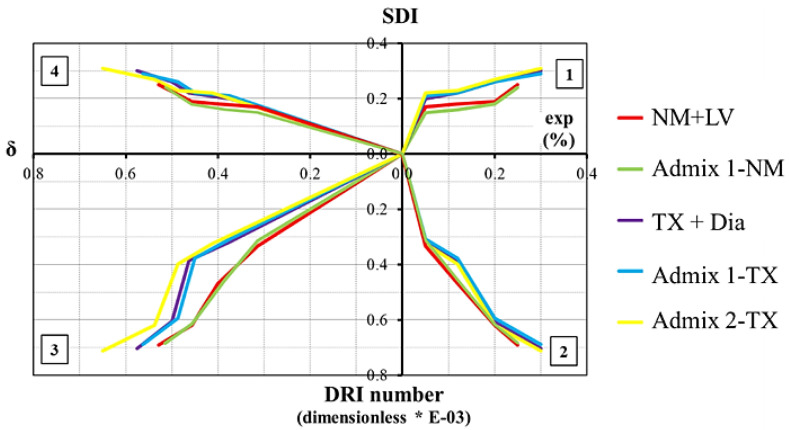
Multilevel assessment chart.

By compiling the data obtained through the use of a comprehensive multilevel assessment (consisting of mechanical SDT and microscopic DRI procedures), a four-quadrant chart could be drawn, as proposed by Sanchez et al. [6] (Figure 8). The chart demonstrates the ASR-induced damage development of various concrete mixtures in this study as a function of expansion level. The latter correlates the expansion level of the concrete specimens (right-wing *x*-axis) with the data gathered through the multilevel assessment: (a) SDI, demonstrating the extent of the physical integrity of concrete [6] (positive *y*-axis), (b) DRI number/1000, addressing the level of microscopic deterioration [6] (negative *y*-axis), and (c) damage variable “δ”, representing the given mechanical property loss of the damaged concrete [6]; modulus of elasticity was selected in this study (negative *x*-axis). Furthermore, the results gathered in this work are also summarized in Table 5. When evaluating the aforementioned charts and table, one observes that although all the mechanical property losses and DRI numbers are within the typical range of values for CC per degree of damage, as displayed in Table 5, both of the mixtures that incorporated the various superplasticizers (i.e., regardless of aggregate types) presented results in the lower and upper ranges using Admixtures 1 and 2, respectively. Moreover, with the influence of Admixture 1 on ASR expansion being lower, this could indicate the mitigation potential of this admixture with a low alkali content, as observed by other researchers and guidelines [34,62].

### 6.2. Validation of the Impact of SPs on ASR-Induced Deterioration

**Figure 9:**
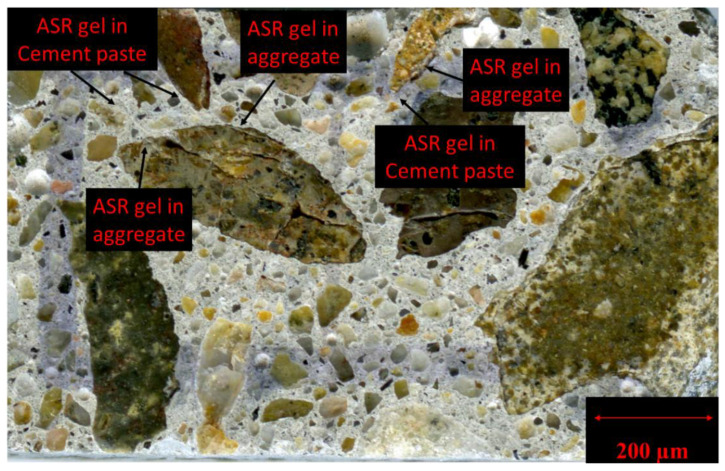
Polished concrete section from the conventional concrete specimens incorporating NM + LV with aggregate and cement paste cracks filled with ASR gel.

In order to validate the results discussed in the previous section, this section investigates the effects of different superplasticizers on the chemical composition of ASR gel and, ultimately, on the extent of the damage to the affected concrete through the use of scanning electron microscopy (SEM) and energy-dispersive X-ray spectroscopy (EDS). Thus, by following the DRI procedure, the concrete specimens with the highest expansion levels in this study (i.e., 0.25% and 0.30% for the NM- and TX-incorporated specimens, respectively) and with large cracks filled with ASR products (e.g., Figure 9—NM + LV at 0.25% of expansion) were identified and further prepared for SEM-EDS analysis. Later, the cut specimens were polished, as per Leemann and Lura [68], and then the polished specimens were examined using scanning electron microscopy (SEM). The SEM device used was a JEOL 6610LV (JEOL, University of Ottawa, Ottawa, ON, Canada) using backscattered electron (BEC) imaging with the operating conditions set at 15 kV and a beam current of 200–220 mA. Finally, the reaction product (i.e., ASR gel) was investigated with energy-dispersive X-ray spectroscopy (EDS). It is worth noting that the concrete specimens were placed at 50 °C for 24 h, followed by the application of a thin layer of Au-Pd prior to conducting SEM/EDS analysis.

**Figure 10:**
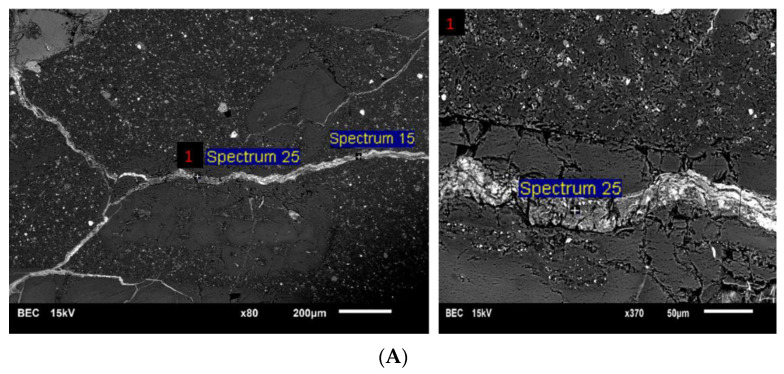

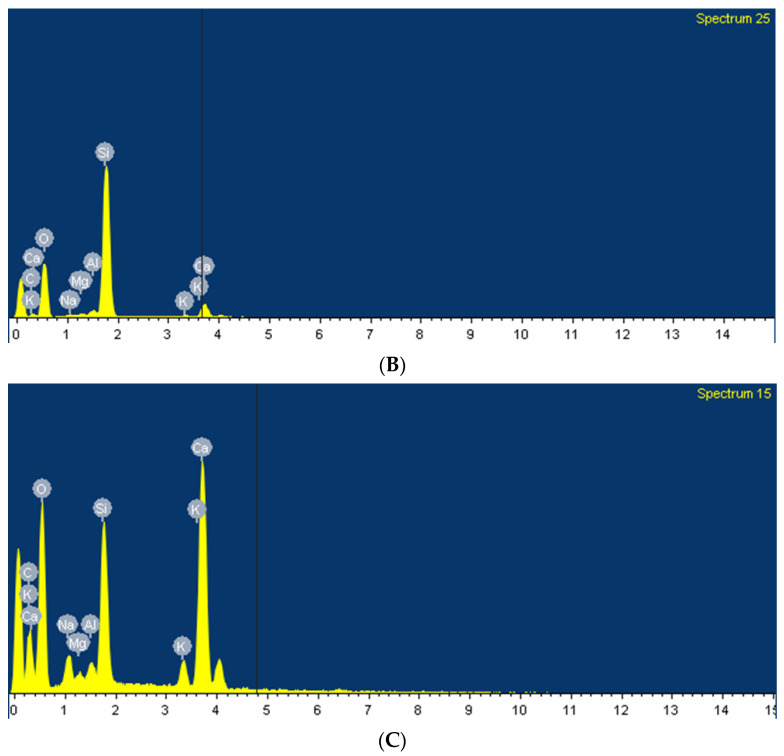
(**A**) SEM image of the polished section of NM + LV (part of Figure 3) with one magnified sector; typical results of the SEM-EDS chemical analysis of the ASR products found in the (**B**) aggregates and (**C**) cement paste.

Figure 10A displays an SEM-BEC image of an aggregate particle from CC made with the reactive coarse aggregate (NM + Lav). Moreover, Figure 10B,C present the samples from the chemical analysis (EDS spectra) for the ASR gel found in the aggregate particles (i.e., silica-rich) and cement paste (i.e., calcium-rich), respectively. Table 6 presents the range of the results obtained from the various points analyzed through the use of EDS for each concrete mixture (i.e., the number of the analyzed points for each concrete mixture can be found in Table 6). The gel’s chemical compositions are in accordance with what has been previously observed by a number of researchers [68,69,70].

**Figure 11:**
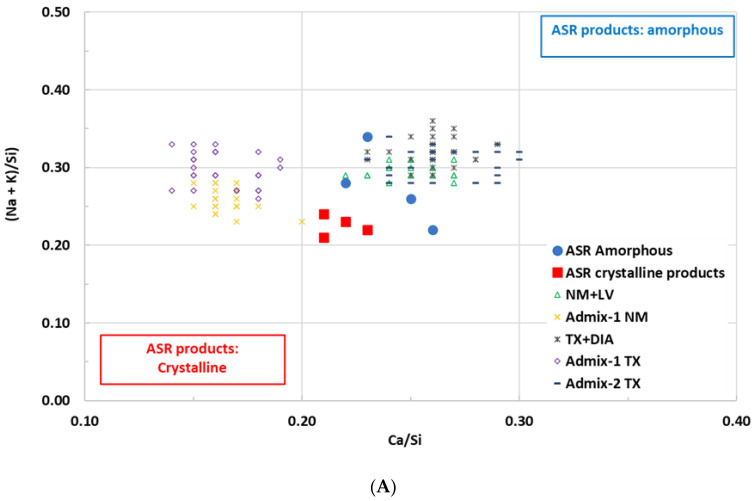

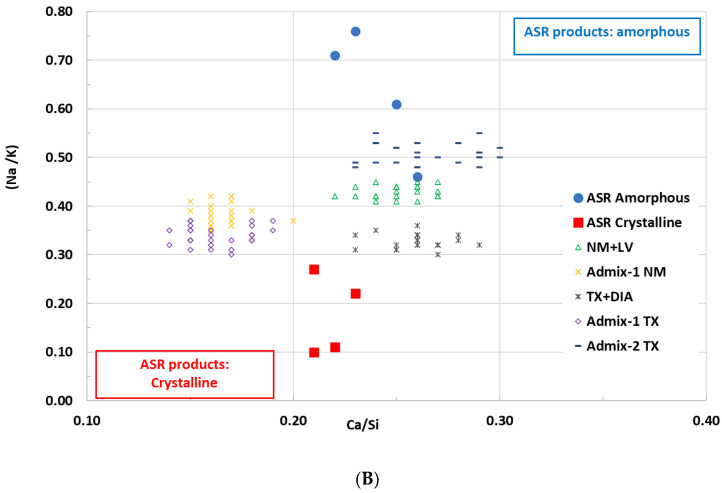
(**A**) The ratio of (Na + K)/Si in the ASR products over the Ca/Si-ratio and (**B**) the ratio of Na/K in the ASR products over the Ca/Si-ratio in comparison with the results gathered by Leemann et al. [72].

Based on the method followed by Ahmed et al. [71], the chemical composition of the ASR gel obtained from the aggregate particles of various concrete types in this work (i.e., as presented in Table 6A) was compared with the typical chemical composition of ASR gel observed by Leemann et al. [72] (displayed in Figure 11). When analyzing the graphs, one sees that the chemical composition of the ASR secondary product found in conventional mixtures (i.e., NM + Lav and TX + Dia), as well as those obtained from Admix 2-TX, are almost in the range from crystalline to amorphous in the proposed composition [72]. On the other hand, when evaluating the chemical composition of the ASR gel gathered from both the concrete mixtures that incorporated Admixture 1 (i.e., the SP with a lower alkali content), it is noted that these ASR secondary products are not quite similar to typical crystalline or amorphous ASR gels, as proposed by Leemann et al. [72]. This observation might be attributed to the low alkali content in the added superplasticizer, and, once again, this attests to the fact that incorporating an admixture with a significantly low alkali content into concrete might mitigate ASR-induced development.

**Figure 12:**
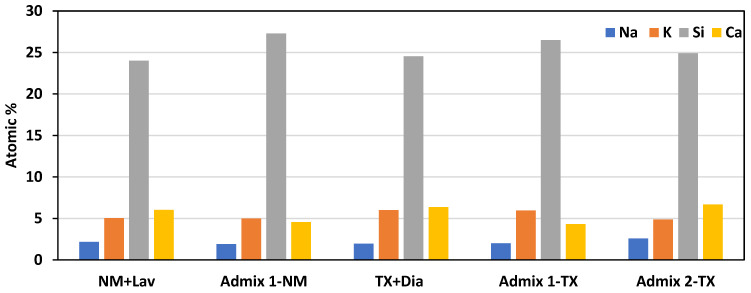
Chemical composition of ASR products (gel) inside the aggregate particles for all concrete mixtures in this study.

As per Table 6A and Figure 11, it is obvious that the composition of the ASR products generated within the reactive aggregate particles of the distinct concrete mixtures varies to a considerable extent. In order to have a better overview, Figure 12 illustrates the key elements of the ASR gel (i.e., an average of all the points per concrete mixture) found in the aggregate particles. When comparing the chemical composition of the latter, obtained from the distinct concrete specimens made with reactive coarse aggregate (i.e., NM), one sees that, while the potassium content of the ASR gels found in CC and Admix 1-NM are almost similar, the Na and Ca content in the ASR gel found in CC is slightly higher than that found in Admix 1-NM (12% and 0.25%, respectively). Conversely, the Si content of the ASR product obtained from Admix 1-NM is 12% higher than that gathered from CC-NM concrete (i.e., K: Admix 1-NM ≈ CC-NM, Na and Ca: Admix 1-NM < CC-NM, and Si: Admix 1-NM > CC-NM). Similarly, although the Na and K concentrations in CC-TX and Admix 1-TX are almost identical, the Ca and Si concentrations in the TX + Dia mixtures without any SP are 32% higher and 7% lower than the ones with the Admixture 1, respectively (i.e., K and Na: Admix 1-TX ≈ CC-TX, Ca: Admix 1-TX < CC-TX, and Si: Admix 1-TC > CC-TX). The lower alkali and Ca content in the ASR gel gathered from the aggregate particles of the concrete specimens with Admixture 1 incorporated (when compared to the conventional concrete samples) could confirm the mitigation potential of it against ASR deterioration. This is in accordance with the results obtained from the induced expansion (Figure 3), mechanical degradation (Figure 4), and microscopic damage assessment (i.e., Figure 5, Figure 6 and Figure 7), which, once again, show that the SPs with a significantly low alkali content can reduce ASR-induced development. Conversely, when comparing the chemical composition of the ASR gel found in the aggregate of the TX + Dia mixtures without and with Admixture 2, one notices that the Na and Ca content of the ASR gel obtained from Admix 2-TX is slightly higher than the conventional mixture (i.e., 24% and 5%, respectively). The higher concentration of Na and Ca in the ASR gels obtained from Admix 2-TX clearly attest to the fact that these ASR products are more deleterious when compared to those gathered from the TX + Dia mixtures without any admixture, as per Sun et al. [73]. The above, once again, is in accordance with the ASR-induced development results gathered throughout this work, where the higher deleterious potential of Admix 2-TX has been observed in comparison to the other concrete mixtures in this work; this could be attributable to the high alkali content in Admixture 2 (i.e., 4.1 ± 0.1% Na_2_O_eq_—Table 2).

**Figure 13:**
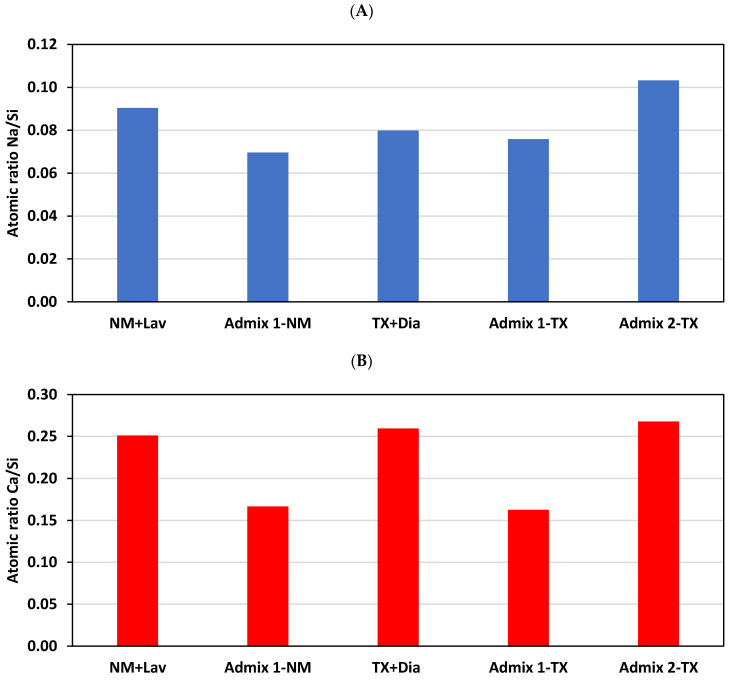
(**A**) Na/Si and (**B**) Ca/Si—ratios of the ASR product (gel) inside the aggregate particles for all concrete mixtures in this study.

In order to comprehensively understand the impact of various SPs on ASR gel properties, Figure 13A,B illustrate the atomic ratio of Na/Si and Ca/Si, respectively, for the ASR products found in the aggregate particles of the various concrete mixtures in this study. As per to Gholizadeh et al. [74] and Sun et al. [73], those ASR gels with a high Na/Si ratio have a higher potential for deterioration (i.e., high expansibility), and those ASR products with a Ca/Si ratio of higher than 0.23 are more prone to deterioration. When evaluating the Na/Si and Ca/Si ratio of the ASR products formed in the aggregate particles of the distinct concrete mixtures, one confirms that those gathered from the Admixture 2- incorporated specimens have the highest potential for further deterioration (i.e., the highest Na/Si ratio: 0.10; highest Ca/Si ratio: 0.27). Conversely, the ASR product obtained from the specimens made with Admixture 1 exhibited the lowest Na/Si (i.e., 0.07 and 0.08 for the concrete specimens manufactured with NM and TX, respectively) and Ca/Si (i.e., 0.17 and 0.16 for NM- and TX-incorporated specimens, respectively) ratio. The above discussion, once again, clearly confirms the mitigation potential of Admixture 1 against ASR deterioration, while Admixture 2 can increase ASR-induced deterioration.

**Figure 14:**
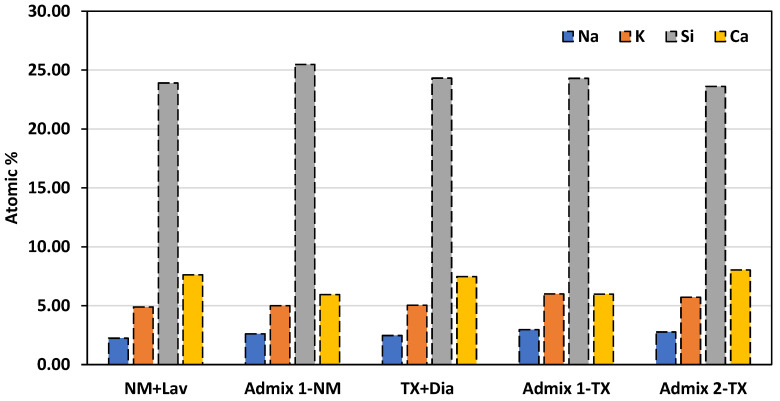
Chemical composition of ASR product (gel) inside the cement paste for all concrete mixtures in this study.

Furthermore, in order to enhance our understanding of the compositions of the ASR gels in the cement paste presented in Table 6, similar to the previous section, the key elements of the ASR gel found in the cement paste (i.e., an average of all points per concrete mixture) in all the concrete mixtures in this study was analyzed (Figure 14). When evaluating the plots, one sees that the ASR gel found in the cement paste of the Admix 2-TX concrete specimens has the highest Ca content among the others, while both the Admixture 1-incorporated concrete specimens (i.e., Admix 1-NM and Admix 1-TX) exhibited the lowest Ca content. When considering the suggestion made by Sun et al. [73], where the authors [73] observed that the higher the Ca content of ASR gel in cement paste, the higher the deleterious potential of the latter, the above discussion, once again, confirms the deterioration and mitigation potential of Admixtures 2 and 1 against ASR, respectively.

## 7. Conclusions

This work intended to investigate the impact of distinct SPs with various alkali content (i.e., Admixtures 1 and 2: 0.00009% and 4.1% Na_2_O_eq_, respectively) on ASR development in concrete specimens made with different aggregate types (i.e., reactive fine and coarse aggregate) via the use of a multilevel assessment. The latter contains a series of mechanical and microscopic techniques, mainly the stiffness damage test (SDT) and the damage rating index (DRI), which have previously been used as reliable techniques in condition assessments of alkali-silica reaction (ASR)-affected concrete. Thus, the main results gathered throughout this study are summarized hereafter:

- The concrete specimens that incorporated Admixture 2 (i.e., 4.1% of Na_2_O_eq_) demonstrated faster ASR-induced expansion, while those made with Admixture 1 (i.e., 0.00009% of Na_2_O_eq_) displayed slower ASR kinetics when compared to the conventional concrete samples. This is likely due to the chemical composition (i.e., alkali content) of the incorporated superplasticizers; the higher the alkali content of the SP used in the mixture, the higher the alkalinity of the pore solution, likely resulting in higher ASR kinetics;
- Similar to expansion behavior, the Admixtures 2 and 1 concrete specimens displayed greater and lesser ASR damage development, respectively. This clearly attests to the effect of alkali content (in distinct SPs) on the development of ASR-induced damage; the higher the alkali content of the SP used in the concrete mixture, the higher the ASR development. Moreover, very low alkali content in an SP used in a concrete mixture can act as a mitigation strategy against ASR development. Nevertheless, the multilevel assessment used in this study proved to be a suitable diagnostic approach that is able to capture the differences in the damage induced by ASR;
- An investigation into the chemical composition of the ASR gel found in the aggregate particles of the distinct concrete specimens showed that the specimens manufactured with Admixture 2 had the highest Na and Ca content, while those incorporating Admixture 1 had the lowest Na and Ca content. This could be a clear sign that the ASR gel analyzed in the aggregate particles of the specimens made with Admixture 2 had the highest ASR deterioration potential. Likewise, the significantly higher Ca content in the ASR gel obtained from the cement paste of the Admix 2 concrete specimens, once again, attests to the higher risk of ASR damage when using the Admixture 2 samples;
- A comparison of the distinct atomic ratios of the various ASR gels (i.e., Na/Si and Ca/Si) gathered from the aggregate particles of the distinct concrete specimens showed that those made with Admixture 2 had the highest Na/Si and Ca/Si ratios, while those incorporating Admixture 1 had the lowest Na/Si and Ca/Si ratios. According to the previous works, this observation clearly attests to the mitigation potential of SP admixtures with very low alkali content against ASR deterioration, while those superplasticizers with high alkali content increase the likelihood of ASR damage.

## Figures and Tables

**Table 1:** Distinct aggregates used in this work, including their physical properties.

Aggregate				Rock Type	Specific Gravity (g/cm^3^)	Absorption (%)	AMBT * (14 d exp)
Type	Reactivity	Location	Name				
Coarse	Reactive	New Mexico (USA)	NM	Polymictic gravel	2.53	1.59	1.056% [54]
	Nonreactive	Québec (Canada)	Dia	Diabase (plutonic rock)	3.00	0.51	0.065% [6]
Fine	Reactive	El Paso (USA)	TX	Polymictic sand	2.60	0.55	0.755% [6]
	Nonreactive	Laval (Canada)	Lv	Natural derived from granite	2.71	0.54	0.068% [6]

* Accelerated mortar bar test (AMBT), as per ASTM C1260 [55].

**Table 2:** Composition of SP admixtures used in this study.

Name	Chemical Composition				pH	Standard Conformity
	Na	K	Na_2_O_eq_	Cl		
Admixture 1	0.8 ppm (0.00008%)	0.2 ppm (0.00002%)	0.9 ppm (0.00009%)	1.6 ppm (0.00016%)	6.5	ASTM C 494, Types F
Admixture 2	4.1 ± 0.1%.	0%	4.1 ± 0.1%.	<0.1%	7.5	ASTM C 494, Types A & F

**Table 3:** Concrete mixtures used in this study.

Materials	Quantities (kg/m^3^)				
	NM + Lav	Admix 1-NM	TX + Dia	Admix 1-TX	Admix 2-TX
Cement	424	424	424	424	424
Fine aggregate (<4.75 mm)	714	714	896	896	896
Coarse aggregate (4.75–19 mm)	1073	1073	1029	1029	1029
Water	157	157	157	157	157

**Table 4:** Two-variable ANOVA using SDI, PDI, and ME.

ANOVA Analysis				SDI								
Specimens type	Load (%)	Expansion (%)		SDI-F	SDI-Fcritic		F > Fcritic	SDI_*p* value		α	*p* < α	
NM + Lav	40	0.05–0.30%		21.94	4.07		✓	0.000324		0.05	✓	
Admix 1-NM	40	0.05–0.30%		39.50	4.07		✓	3.84 × 10^−5^		0.05	✓	
TX + Dia	40	0.05–0.30%		19.13	4.07		✓	0.000523		0.05	✓	
Admix 1-TX	40	0.05–0.30%		31.84	4.07		✓	8.5 × 10^−5^		0.05	✓	
Admix 2-TX	40	0.05–0.30%		35.87	4.07		✓	5.49 × 10^−5^		0.05	✓	
ANOVA analysis	PDI						ME					
Specimens type	PDI-F	PDI-Fcritic	F > Fcritic	PDI_*p* value	α	*p* < α	ME-F	ME-Fcritic	F > Fcritic	ME_*p* value	α	*p* < α
NM + Lav	25.31	4.07	✓	0.000195	0.05	✓	67.49	4.07	✓	5.06 × 10^−6^	0.05	✓
Admix 1-NM	11.27	4.07	✓	0.003038	0.05	✓	38.70	4.07	✓	4.14 × 10^−5^	0.05	✓
TX + Dia	14.04	4.07	✓	0.001493	0.05	✓	53.76	4.07	✓	1.2 × 10^−5^	0.05	✓
Admix 1-TX	6.85	4.07	✓	0.013342	0.05	✓	62.91	4.07	✓	6.62 × 10^−6^	0.05	✓
Admix 2-TX	67.89	4.07	✓	4.95 × 10^−6^	0.05	✓	40.28	4.07	✓	3.57 × 10^−5^	0.05	✓

**Table 5:** Comparison of damage classification.

Classification of ASR Damage Degree (%)	Reference Expansion Level (%)	Assessment of ASR								
		Conventional Concrete			Concrete Specimens Incorporating SP with Low Alkali			Concrete Specimens Incorporating SP with High Alkali		
		ME Loss (%)	SDI	DRI	ME Loss (%)	SDI	DRI	ME Loss (%)	SDI	DRI
Negligible	0.00–0.03	-	0.06–0.16	100–155	-	0.08	130–140	-	0.08–0.09	130–145
Marginal	0.04 ± 0.01	5–37	0.11–0.25	210–400	30–38	0.15–0.21	300–315	38–41	0.20–0.22	320–335
Moderate	0.11 ± 0.01	20–50	0.15–0.31	330–500	39–45	0.16–0.22	370–450	45–50	0.23–0.24	390–405
High	0.20 ± 0.01	35–60	0.19–0.32	500–765	46–49	0.18–0.26	590–615	50–55	0.26–0.28	600–625
Very high	0.30 ± 0.01	40–67	0.22–0.36	600–925	50–57	0.24–0.29	680–690	60–65	0.30–0.32	700–720

**Table 6:** Gel composition of cracks in (A) aggregate particles and (B) cement paste.

**A**													
**Mixtures**	**Source of ASR Products**	**Elements in Atomic-%**											**Number of Points**
		**Na**	**Mg**	**Si**	**S**	**K**	**Ca**	**O**	**Ca/Si**	**(Na + K)/Si**	**Na/Si**	**Na/K**	
NM + Lav	Aggregate particles	2.17 ± 0.8	0.05 ± 0.02	24.01 ± 4.45	0.00 ± 0.00	5.04 ± 1.3	6.03 ± 1.6	61.15 ± 3.2	0.25 ± 0.02	0.30 ± 0.01	0.09 ± 0.02	0.43 ± 0.04	53
Admix 1-NM	Aggregate particles	1.90 ± 0.22	0.05 ± 0.05	27.30 ± 4.22	0.00 ± 0.00	4.99 ± 0.52	4.55 ± 1.11	61.09 ± 4.23	0.16 ± 0.02	0.25 ± 0.02	0.07 ± 0.02	0.38 ± 0.05	62
TX + DIA	Aggregate particles	1.96 ± 0.7	0.05 ± 0.01	24.55 ± 3.11	0.02 ± 0.01	6.01 ± 1.46	6.37 ± 1.02	60.01 ± 4.11	0.26 ± 0.12	0.32 ± 0.02	0.08 ± 0.01	0.32 ± 0.04	46
Admix 1-TX	Aggregate particles	2.01 ± 0.10	0.09 ± 0.05	26.50 ± 3.89	0.01 ± 0.01	5.95 ± 0.85	4.31 ± 1.20	60.18 ± 4.11	0.16 ± 0.02	0.30 ± 0.1	0.07 ± 0.02	0.33 ± 0.03	36
Admix 2-TX	Aggregate particles	2.57 ± 0.22	0.09 ± 0.07	24.90 ± 3.10	0.00 ± 0.00	4.88 ± 0.88	6.67 ± 1.10	60.01 ± 2.01	0.26 ± 0.05	0.30 ± 0.04	0.10 ± 0.7	0.52 ± 0.06	25
**B**													
**Mixtures**	**Source of ASR Products**	**Elements in Atomic-%**											**Number of Points**
		**Na**	**Mg**	**Si**	**S**	**K**	**Ca**	**O**	**Ca/Si**	**(Na + K)/Si**	**Na/Si**	**Na/K**	
NM + Lav	Cement Paste	2.24 ± 0.44	0.02 ± 0.01	23.901 ± 2.11	0.00 ± 0.01	4.89 ± 0.21	7.62 ± 0.6	61.07 ± 1.82	0.32 ± 0.21	0.29 ± 0.02	0.09 ± 0.01	0.45 ± 0.02	40
Admix 1-NM	Cement Paste	2.61 ± 0.21	0.03 ± 0.01	25.48 ± 2.36	0.02 ± 0.02	4.99 ± 0.44	5.94 ± 0.66	60.48 ± 2.85	0.23 ± 0.36	0.29 ± 0.01	0.10 ± 0.01	0.52 ± 0.01	40
TX + DIA	Cement Paste	2.47 ± 0.14	0.02 ± 0.01	24.32 ± 1.25	0.00 ± 0.01	5.03 ± 0.36	7.42 ± 0.54	60.52 ± 2.88	0.30 ± 0.36	0.30 ± 0.02	0.10 ± 0.01	0.49 ± 0.03	40
Admix 1-TX	Cement Paste	2.97 ± 0.14	0.02 ± 0.01	24.30 ± 2.11	0.00 ± 0.02	5.99 ± 0.44	5.98 ± 0.56	59.85 ± 2.10	0.24 ± 0.52	0.36 ± 0.02	0.12 ± 0.02	0.49 ± 0.02	30
Admix 2-TX	Cement Paste	2.77 ± 0.66	0.03 ± 0.01	23.61 ± 3.01	0.01 ± 0.01	5.72 ± 42	8.03 ± 0.42	59.52 ± 2.02	0.34 ± 0.14	0.35 ± 0.02	0.11 ± 0.02	0.48 ± 0.01	39

## Data Availability

Not applicable.
